# Diagnostic accuracy of endoscopic ultrasound, computed tomography, magnetic resonance imaging, and endorectal ultrasonography for detecting lymph node involvement in patients with rectal cancer: A protocol for an overview of systematic reviews

**DOI:** 10.1097/MD.0000000000012899

**Published:** 2018-10-26

**Authors:** Xin Wang, Ya Gao, Jipin Li, Jiarui Wu, Bo Wang, Xueni Ma, Jinhui Tian, Minghui Shen, Jiancheng Wang

**Affiliations:** a Department of Ultrasound Medicine, Second Hospital of Lanzhou University; b Evidence-Based Medicine Center, School of Basic Medical Sciences, Lanzhou University; c The Second Clinical Medical College of Lanzhou University, Lanzhou; d Department of Clinical Pharmacology of Traditional Chinese Medicine, School of Chinese Materia Medica, Beijing University of Chinese Medicine, Beijing; e Department of Nursing, Rehabilitation Center Hospital of Gansu Province; f Department of Clinical Laboratory, Second Hospital of Lanzhou University; g Gansu Provincial Hospital, Lanzhou, China.

**Keywords:** computed tomography, diagnostic test, endorectal ultrasonography, endoscopic ultrasound, lymph node involvement, magnetic resonance imaging, overview, rectal cancer

## Abstract

**Background:**

Rectal cancer is one of the most common tumors and is the leading cause of cancer-related deaths in developed countries. Lymph node involvement remains the strongest prognostic factor associated with a worse prognosis in patients with rectal cancer. Several systematic reviews have investigated the accuracy of endoscopic ultrasound, computed tomography, magnetic resonance imaging, and endorectal ultrasonography for lymph node involvement of rectal cancer and compared the diagnostic accuracy of different imaging techniques, but there are considerable differences in conclusions. This study aims to assess the methodological quality and reporting quality of systematic reviews and to determine which diagnostic imaging techniques is the optimal modality for the diagnosis of lymph node involvement in patients with rectal cancer.

**Methods:**

We will search PubMed, EMBASE, Cochrane Library, and Chinese Biomedicine Literature to identify relevant studies from inception to June 2018. We will include systematic reviews that evaluated the accuracy of diagnostic imaging techniques for lymph node involvement. The methodological quality will be assessed using AMASAR checklist, and the reporting quality will be assessed using PRISMA-DTA checklist. The pairwise meta-analysis and indirect comparisons will be performed using STATA V.12.0.

**Results:**

The results of this overview will be submitted to a peer-reviewed journal for publication.

**Conclusion:**

This overview will provide comprehensive evidence of different diagnostic imaging techniques for detecting lymph node involvement in patients with rectal cancer.

**Ethics and dissemination:**

Ethics approval and patient consent are not required as this study is an overview based on published systematic reviews.

**PROSPERO registration number:**

CRD42018104906.

## 1 Introduction

Colorectal cancer is nowadays the third most common cancer in men and the second in women worldwide, accounting for 9% of new cancer cases and 9% of cancer-specific deaths.^[1,2]^ Approximately 1 in 3 of these tumors are rectal cancers.^[3]^ In the USA, rectal cancer is a major cause of mortality, and there were an estimated 39,220 new cases in 2016.^[4]^ Risk factors for rectal cancer include familial polyposis syndromes (FAP, HNPCC), history of adenomatous polyps, diabetes mellitus, obesity, excessive alcohol, and cigarette smoking.^[5–10]^ Because of the diffusion of screening programs, the incidence of this malignancy has gradually increased.^[11,12]^

As in most solid malignancies, lymph node involvement remains the strongest prognostic factor associated with a worse prognosis in patients with rectal cancer.^[13,14]^ However, the prognosis of rectal cancer patients depends on the disease stage at the time of diagnosis; thus, accurate disease evaluation is necessary to properly treat rectal cancer.^[15–17]^ Nowadays, treatment of rectal cancer with different locations and stages mainly includes transanal endoscopic microsurgery, anterior resection with total mesorectal excision, abdominoperineal resection, and neoadjuvant chemotherapy and radiotherapy.^[2,18,19]^ Accurate preoperative assessment of lymph node involvement is essential for selecting patients to receive optimal treatment.^[2,20]^

Various modalities, endoscopic ultrasound (EUS), computed tomography (CT), magnetic resonance imaging (MRI), and endorectal ultrasonography (ERUS), for instance, have been used to assess the lymph node status. EUS is particularly effective for assessing the depth of tumor invasion into the rectal wall.^[21]^ CT is a sensitive method for diagnosis of abdominal and pelvic diseases. It is used frequently to determine the stage of cancer and to follow progress.^[14]^ MRI allows for comprehensive evaluation of disease stage including tumor infiltration degree, a precise assessment of the neoplasia distance by mesorectal fascia (circumferential margin) and an effective assessment of lymph nodes involvement and mesorectal infiltration.^[22,23]^ ERUS is considered to be most accurate for small tumors (tumor stages 1 and 2).^[24]^

Recently, some meta-analysis has investigated the accuracy of EUS, CT, MRI, and ERUS for lymph node involvement of rectal cancer and compared the diagnostic accuracy of different imaging techniques,^[3,25–27]^ but there are considerable differences in conclusions. Therefore, it is of great significance to re-evaluate these systematic reviews. The objectives of this overview are to assess the methodological quality and reporting quality of systematic reviews that evaluated the diagnostic value of index tests for lymph node involvement in patients with rectal cancer and to compare the diagnostic value of different diagnostic imaging techniques for lymph node involvement by reanalyzing the results of meta-analysis.

## 2 Methods

### 2.1 Design and registration

We will conduct an overview of systematic reviews of diagnostic test accuracy. The protocol is registered in PROSPERO (CRD42018104906). We will follow the Preferred Reporting Items for Systematic Reviews and Meta-analysis^[28]^ statements for reporting our overview.

### 2.2 Eligibility criteria

#### 2.2.1 Type of study

Systematic reviews will meet the following criteria: diagnostic imaging techniques include EUS, CT, MRI, and ERUS or combinations; evaluate the diagnostic value of index tests for lymph node involvement in patients with rectal cancer. Systematic reviews for patients with colorectal cancer will be excluded.

#### 2.2.2 Patients

We will include rectal cancer patients with lymph node involvement regardless of treatment. No limitations will be imposed on age, sex, or nationality.

#### 2.2.3 Interventions

We will regard EUS, CT, MRI, or ERUS as index tests because these tests are usually used to predict lymph node involvement in patients with rectal cancer. In addition, other index tests for detecting lymph node involvement are also included.

#### 2.2.4 Outcomes

The primary outcomes are sensitivity (SEN), specificity (SPE), positive predictive value, negative predictive value, positive likelihood ratio (PLR), negative likelihood ratio (NLR), diagnostic odds ratio (DOR), area under the curve, and their respective 95% confidence intervals (CIs). The second outcomes are methodological quality score and reporting quality score.

### 2.3 Data sources

We will search PubMed, EMBASE, Cochrane Library, and Chinese Biomedicine Literature to identify relevant studies from inception to June 2018. Publication languages will be restricted to English and Chinese. In addition, we will check reference lists of included studies for additional references.

### 2.4 Search strategy

We will use search terms related to rectal neoplasm, SEN, SPE, receiver operating characteristic, meta-analysis, and systematic review. Search strategy of PubMed was as follows:

#1 “Rectal Neoplasms”[Mesh]
#2 “rect∗ neoplasm∗”[Title/Abstract] OR “rect∗ canc∗”[Title/Abstract] OR “rect∗ carcinom∗”[Title/Abstract] OR “rect∗ adenocarc∗”[Title/Abstract] OR “rect∗ tumor∗”[Title/Abstract] OR “rect∗ tumour∗”[Title/Abstract] OR “rect∗ sarcom∗”[Title/Abstract]
#3 #1 OR #2
#4 “Sensitivity AND Specificity”[Mesh] OR “False Positive Reactions”[Mesh] OR “False Negative Reactions”[Mesh] OR “ROC Curve”[Mesh] OR “Predictive Value of Tests”[Mesh]
#5 sensitivity[Title/Abstract] OR specificity[Title/Abstract] OR receiver operating characteristic[Title/Abstract] OR receiver operator characteristic[Title/Abstract] OR predictive value∗[Title/Abstract] OR roc[Title/Abstract] OR pre-test odds[Title/Abstract] OR pretest odds[Title/Abstract] OR pre-test probability∗[Title/Abstract] OR pretest probability∗[Title/Abstract] OR post-test odds[Title/Abstract] OR posttest odds[Title/Abstract] OR post test probabilit∗[Title/Abstract] OR posttest probabilit∗ [Title/Abstract] OR likelihood ratio∗[Title/Abstract] OR positive predictive value∗[Title/Abstract] OR negative predictive value∗[Title/Abstract] OR false negative∗[Title/Abstract] OR false positive∗[Title Abstract] OR true negative∗ [Title/Abstract] OR true positive∗[Title/Abstract] OR fn[Title/Abstract] OR fp[Title/Abstract] OR tn[Title/Abstract] OR tp [Title/Abstract]
#6 #4 OR #5
#7 “Meta-Analysis” [Publication Type] OR “Meta-Analysis as Topic”[Mesh]
#8 Meta-Analysis[Title/Abstract] OR Meta-Analyses[Title/Abstract] OR Meta Analysis[Title/Abstract] OR Meta Analyses[Title/Abstract] OR gathering analysis[Title/Abstract]
#9 “Network Meta-Analysis”[Mesh]
#10 Network Meta-Analyses[Title/Abstract] OR Network Meta Analysis[Title/Abstract] OR Network Meta Analyses[Title/Abstract] OR Mixed Treatment Meta-Analysis[Title/Abstract] OR Mixed Treatment Meta-Analyses[Title/Abstract] OR Multiple Treatment Comparison Meta-Analysis[Title/Abstract] OR Multiple Treatment Comparison Meta Analysis[Title/Abstract]
#11 Systematic evaluation[Title/Abstract] OR Systematic assessment[Title/Abstract] OR Systematic review[Title/Abstract] OR Systematic reviews[Title/Abstract] OR System evaluation[Title/Abstract] OR System Assessment[Title/Abstract] OR Systemic review[Title/Abstract] OR Systemic reviews[Title/Abstract]
#12 #7 OR #8 OR #9 OR #10 OR #11
#13 #3 AND #6 AND #12

### 2.5 Study selection and data extraction

Literature search records will be imported into ENDNOTE X7 literature management software. Two independent reviewers will screen out possibly relevant studies independently based on the title and abstract. Then, the same 2 reviewers will retrieve the full text of all possibly relevant studies to screen out the studies that meet the inclusion criteria. We will extract study characteristics from systematic reviews including the following items: author name, year of publication, country of first author, number of author, journal name, country of journal, funding, disease, number and name of index test, number and name of reference test, outcomes; methodological characteristics of systematic reviews such as types of included studies, number of included studies, samples, number and name of databases retrieved, supplemental literature search; and results of statistical analysis including SEN, SPE, likelihood ratio, predictive value, DOR, and area under curve. Disagreements will be resolved by consensus or by discussion with a third reviewer.

### 2.6 Quality assessment

We will assess the methodological quality of included systematic reviews using the Assessment of Multiple Systematic Reviews checklist. This checklist includes 11 items with scores ranging from 0 to 11.0. Based on previous overviews, we will consider studies with a score between 0 and 4.0 to be of low quality, 5.0 and 8.0 to be of moderate quality, and 9.0 and 11.0 to be of high quality.^[29,30]^

The reporting quality of included systematic reviews will be assessed according to the Preferred Reporting Items for Systematic Reviews and Meta-analysis diagnostic test accuracy (PRISMA-DTA) checklist. The PRISMA-DTA statement is an expanded checklist of original PRISMA, which aims to improve the completeness and transparency of reporting of systematic reviews of diagnostic test accuracy studies.^[31]^ This checklist consists of 27 items. The maximum score on the PRISMA-DTA is 27. The review will be considered to have major flaws if it receives a total score of ≤15.0, minor flaws if it receives a total score of 15.5 to 21.0, and minimal flaws if it receives a total score 21.5 to 27.0.^[32]^ The quality assessment of the included systematic reviews will be performed independently by the 2 authors and the differences will be resolved through discussion to reach a consensus.

### 2.7 Statistical analysis

#### 2.7.1 Pairwise meta-analysis

We will perform pairwise meta-analysis with the data of pooled SEN, SPE, DOR, PLR, NLR, and their 95% CI lower limit, 95% CI upper limit using bivariate mixed-effects regression modeling with STATA V.12.0 (Stata). The between-study variance will be calculated var logitSEN and logitSPE.^[33,34]^ The proportion of heterogeneity due to the threshold effect among the included studies will be calculated by the squared correlation coefficient estimated from the between-study covariance variable in the bivariate model.^[35]^ The heterogeneity between each study will be estimated using the *Q* value and the inconsistency index (*I*^2^) test, and the values of 25%, 50%, and 75% for the *I*^2^ will be indicative of low, moderate, and high statistical heterogeneity, respectively.^[36]^

#### 2.7.2 Indirect comparisons between competing diagnostic tests

We will calculate relative diagnostic outcomes between index tests including relative SEN, relative SPE, relative DOR, relative PLR, and relative NLR. Then, we will conduct indirect comparisons using the relative diagnostic outcomes. All analysis will be performed using Stata software (version 12.0).

#### 2.7.3 Assessment of reporting bias

If there are 10 or more studies in the network meta-analysis, we will use the funnel plot to evaluate the potential publication bias.^[37]^

#### 2.7.4 Subgroup analysis

Subgroup analysis will be conducted according to the difference of time period of index tests, treatment of rectal cancer, MRI field strength, types of EUS, and CT if the necessary data are available.

## 3 Discussion

It is not uncommon to have several systematic reviews under the same topic published evaluating the same interventions, yet without consistent conclusions.^[38]^ However, no overviews summarize evidence for systematic reviews of the diagnostic test for patients with rectal cancer. According to our knowledge, this will be the first overview to assess the methodological quality using AMASAR checklist and the reporting quality using PRISMA-DTA checklist of systematic reviews evaluating the diagnostic value of index tests for lymph node involvement in patients with rectal cancer. Moreover, we will perform indirect comparisons between different diagnostic imaging techniques, which can clearly show the differences between different modalities. We hope that the results of this overview will help clinicians and patients to select an appropriate diagnostic test for rectal cancer.

## Author contributions

XW, YG, and JW planed and designed the research. JL, JW, and BW tested the feasibility of the study. JT, MS, and JW provided methodological advice, polished, and revised the manuscript. XW and YG wrote the manuscript. All authors approved the final version of the manuscript.

**Methodology:** Jiarui Wu, Xueni Ma, Jinhui Tian, Minghui Shen, Jiancheng Wang.

**Project administration:** Xin Wang, Ya Gao, Jiancheng Wang.

**Resources:** Jipin Li, Bo Wang.

**Software:** Jipin Li, Xueni Ma, Jinhui Tian.

**Supervision:** Jiancheng Wang.

**Validation:** Bo Wang.

**Writing – original draft:** Xin Wang, Ya Gao.

**Writing – review and editing:** Jinhui Tian, Jiancheng Wang.

## References

[1] Jemal A Bray F Center MM Global cancer statistics. CA Cancer J Clin 2011;61:69–90. 21296855 10.3322/caac.20107

[2] Zhang G Cai YZ Xu GH Diagnostic accuracy of MRI for assessment of T category and circumferential resection margin involvement in patients with rectal cancer: a meta-analysis. Dis Colon Rectum 2016;59:789–99. 27384098 10.1097/DCR.0000000000000611

[3] Li XT Sun YS Tang L Evaluating local lymph node metastasis with magnetic resonance imaging, endoluminal ultrasound and computed tomography in rectal cancer: a meta-analysis. Colorectal Dis 2015;17:O129–35. 25628186 10.1111/codi.12909

[4] Siegel RL Miller KD Jemal A Cancer statistics, 2016. CA Cancer J Clin 2016;66:7–30. 26742998 10.3322/caac.21332

[5] Puli SR Reddy JB Bechtold ML Accuracy of endoscopic ultrasound to diagnose nodal invasion by rectal cancers: a meta-analysis and systematic review. Ann Surg Oncol 2009;16:1255–65. 19219506 10.1245/s10434-009-0337-4

[6] Atkin WS Morson BC Cuzick J Long-term risk of colorectal cancer after excision of rectosigmoid adenomas. N Engl J Med 1992;326:658–62. 1736104 10.1056/NEJM199203053261002

[7] Larsson SC Orsini N Wolk A Diabetes mellitus and risk of colorectal cancer: a meta-analysis. J Natl Cancer Inst 2005;97:1679–87. 16288121 10.1093/jnci/dji375

[8] Cho E Smith-Warner SA Ritz J Alcohol intake and colorectal cancer: a pooled analysis of 8 cohort studies. Ann Intern Med 2004;140:603–13. 15096331 10.7326/0003-4819-140-8-200404200-00007

[9] Giovannucci E Ascherio A Rimm EB Physical activity, obesity, and risk for colon cancer and adenoma in men. Ann Intern Med 1995;122:327–34. 7847643 10.7326/0003-4819-122-5-199503010-00002

[10] Paskett ED Reeves KW Rohan TE Association between cigarette smoking and colorectal cancer in the Women's Health Initiative. J Natl Cancer Inst 2007;99:1729–35. 18000222 10.1093/jnci/djm176

[11] Delbeke D Martin WH PET and PET-CT for evaluation of colorectal carcinoma. Semin Nucl Med 2004;34:209–23. 15202102 10.1053/j.semnuclmed.2004.03.006

[12] Maffione AM Chondrogiannis S Capirci C Early prediction of response by 18F-FDG PET/CT during preoperative therapy in locally advanced rectal cancer: a systematic review. Eur J Surg Oncol 2014;40:1186–94. 25060221 10.1016/j.ejso.2014.06.005

[13] Kapiteijn E Marijnen CA Nagtegaal ID Preoperative radiotherapy combined with total mesorectal excision for resectable rectal cancer. N Engl J Med 2001;345:638–46. 11547717 10.1056/NEJMoa010580

[14] Sun YS Li XT Tang L Magnetic resonance imaging (MRI) versus computed tomography (CT) for the diagnosis of lymph node metastasis in preoperative rectal cancer. Cochrane Database Syst Rev 2012;5:CD009867.

[15] Heo SH Kim JW Shin SS Multimodal imaging evaluation in staging of rectal cancer. World J Gastroenterol 2014;20:4244–55. 24764662 10.3748/wjg.v20.i15.4244 PMC3989960

[16] Gunderson LL Jessup JM Sargent DJ Revised tumor and node categorization for rectal cancer based on surveillance, epidemiology, and end results and rectal pooled analysis outcomes. J Clin Oncol 2010;28:256–63. 19949015 10.1200/JCO.2009.23.9194 PMC2815714

[17] Benson AB 3rd Bekaii-Saab T Chan E Rectal cancer. J Natl Compr Canc Netw 2012;10:1528–64. 23221790 10.6004/jnccn.2012.0158

[18] Engstrom PF Arnoletti JP Benson AB 3rd NCCN Clinical Practice Guidelines in Oncology: rectal cancer. J Natl Compr Canc Netw 2009;7:838–81. 19755047 10.6004/jnccn.2009.0057

[19] Dinter DJ Hofheinz RD Hartel M Preoperative staging of rectal tumors: comparison of endorectal ultrasound, hydro-CT, and high-resolution endorectal MRI. Onkologie 2008;31:230–5. 18497511 10.1159/000121359

[20] Gagliardi G Bayar S Smith R Preoperative staging of rectal cancer using magnetic resonance imaging with external phase-arrayed coils. Arch Surg 2002;137:447–51. 11926950 10.1001/archsurg.137.4.447

[21] Lin S Luo G Gao X Application of endoscopic sonography in preoperative staging of rectal cancer: six-year experience. J Ultrasound Med 2011;30:1051–7. 21795480 10.7863/jum.2011.30.8.1051

[22] Petrillo A Catalano O Delrio P Post-treatment fistulas in patients with rectal cancer: MRI with rectal superparamagnetic contrast agent. Abdom Imaging 2007;32:328–31. 16969602 10.1007/s00261-006-9028-9

[23] Fusco R Petrillo M Granata V Magnetic resonance imaging evaluation in neoadjuvant therapy of locally advanced rectal cancer: a systematic review. Radiol Oncol 2017;51:252–62. 28959161 10.1515/raon-2017-0032 PMC5611989

[24] Kim SH Lee JM Hong SH Locally advanced rectal cancer: added value of diffusion-weighted MR imaging in the evaluation of tumor response to neoadjuvant chemo- and radiation therapy. Radiology 2009;253:116–25. 19789256 10.1148/radiol.2532090027

[25] De Jong EA Ten Berge JC Dwarkasing RS The accuracy of MRI, endorectal ultrasonography, and computed tomography in predicting the response of locally advanced rectal cancer after preoperative therapy: a metaanalysis. Surgery 2016;159:688–99. 26619929 10.1016/j.surg.2015.10.019

[26] Bipat S Glas AS Slors FJ Rectal cancer: local staging and assessment of lymph node involvement with endoluminal US, CT, and MR imaging--a meta-analysis. Radiology 2004;232:773–83. 15273331 10.1148/radiol.2323031368

[27] Zhao RS Wang H Zhou ZY Restaging of locally advanced rectal cancer with magnetic resonance imaging and endoluminal ultrasound after preoperative chemoradiotherapy: a systemic review and meta-analysis. Dis Colon Rectum 2014;57:388–95. 24509465 10.1097/DCR.0000000000000022

[28] Moher D Liberati A Tetzlaff J Preferred reporting items for systematic reviews and meta-analysis: the PRISMA statement. Int J Surg 2010;8:336–41. 20171303 10.1016/j.ijsu.2010.02.007

[29] Jaspers MW Smeulers M Vermeulen H Effects of clinical decision-support systems on practitioner performance and patient outcomes: a synthesis of high-quality systematic review findings. J Am Med Inform Assoc 2011;18:327–34. 21422100 10.1136/amiajnl-2011-000094 PMC3078663

[30] Monasta L Batty GD Cattaneo A Early-life determinants of overweight and obesity: a review of systematic reviews. Obes Rev 2010;11:695–708. 20331509 10.1111/j.1467-789X.2010.00735.x

[31] McInnes MDF Moher D Thombs BD Preferred reporting items for a systematic review and meta-analysis of diagnostic test accuracy studies: rhe PRISMA-DTA statement. JAMA 2018;319:388–96. 29362800 10.1001/jama.2017.19163

[32] Li JL Ge L Ma JC Quality of reporting of systematic reviews published in “evidence-based” Chinese journals. Syst Rev 2014;3:58. 24906805 10.1186/2046-4053-3-58 PMC4059173

[33] Reitsma JB Glas AS Rutjes AW Bivariate analysis of sensitivity and specificity produces informative summary measures in diagnostic reviews. J Clin Epidemiol 2005;58:982–90. 16168343 10.1016/j.jclinepi.2005.02.022

[34] Higgins JP Thompson SG Deeks JJ Measuring inconsistency in meta-analysis. BMJ 2003;327:557–60. 12958120 10.1136/bmj.327.7414.557 PMC192859

[35] Harbord RM Deeks JJ Egger M A unification of models for meta-analysis of diagnostic accuracy studies. Biostatistics 2007;8:239–51. 16698768 10.1093/biostatistics/kxl004

[36] Ge L Pan B Song F Comparing the diagnostic accuracy of five common tumour biomarkers and CA19-9 for pancreatic cancer: a protocol for a network meta-analysis of diagnostic test accuracy. BMJ Open 2017;7:e018175. 10.1136/bmjopen-2017-018175 PMC5770961 29282264

[37] Deeks JJ Macaskill P Irwig L The performance of tests of publication bias and other sample size effects in systematic reviews of diagnostic test accuracy was assessed. J Clin Epidemiol 2005;58:882–93. 16085191 10.1016/j.jclinepi.2005.01.016

[38] Xing D Wang B Zhang W Intra-articular platelet-rich plasma injections for knee osteoarthritis: an overview of systematic reviews and risk of bias considerations. Int J Rheum Dis 2017;20:1612–30. 29210206 10.1111/1756-185X.13233

